# Hypoxia-Controlled EphA3 Marks a Human Endometrium-Derived Multipotent Mesenchymal Stromal Cell that Supports Vascular Growth

**DOI:** 10.1371/journal.pone.0112106

**Published:** 2014-11-24

**Authors:** Catherine To, Rae H. Farnsworth, Mary E. Vail, Chanly Chheang, Caroline E. Gargett, Carmel Murone, Carmen Llerena, Andrew T. Major, Andrew M. Scott, Peter W. Janes, Martin Lackmann

**Affiliations:** 1 Department of Biochemistry & Molecular Biology, Monash University, Melbourne, Victoria, Australia; 2 Department of Anatomy & Developmental Biology, Monash University, Melbourne, Victoria, Australia; 3 MIMR-PHI Institute for Medical Research, Clayton, Victoria, Australia; 4 Ludwig Institute for Cancer Research, Olivia Newton-John Cancer & Wellness Centre, Melbourne, Victoria, Australia; Center for Molecular Biotechnology, Italy

## Abstract

Eph and ephrin proteins are essential cell guidance cues that orchestrate cell navigation and control cell-cell interactions during developmental tissue patterning, organogenesis and vasculogenesis. They have been extensively studied in animal models of embryogenesis and adult tissue regeneration, but less is known about their expression and function during human tissue and organ regeneration. We discovered the hypoxia inducible factor (HIF)-1α-controlled expression of EphA3, an Eph family member with critical functions during human tumour progression, in the vascularised tissue of regenerating human endometrium and on isolated human endometrial multipotent mesenchymal stromal cells (eMSCs), but not in other highly vascularised human organs. EphA3 affinity-isolation from human biopsy tissue yielded multipotent CD29^+^/CD73^+^/CD90^+^/CD146^+^ eMSCs that can be clonally propagated and respond to EphA3 agonists with EphA3 phosphorylation, cell contraction, cell-cell segregation and directed cell migration. EphA3 silencing significantly inhibited the ability of transplanted eMSCs to support neovascularisation in immunocompromised mice. In accord with established roles of Eph receptors in mediating interactions between endothelial and perivascular stromal cells during mouse development, our findings suggest that HIF-1α-controlled expression of EphA3 on human MSCs functions during the hypoxia-initiated early stages of adult blood vessel formation.

## Introduction

Mammalian tissue growth is controlled by oxygen and nutrient supply, where hypoxia inducible transcription factors (HIFs) respond to oxygen depletion by activating gene programs that initiate the formation and/or expansion of vascular networks [1], [2]. Endothelial cells, endothelial progenitor cells and mural cells that are recruited locally and from the bone marrow are coordinately assembled into functional blood vessels, contributing to the luminal endothelial lining and the supporting perivascular or mural layer [3]–[5]. Notably, considerable evidence is accumulating for the involvement of multipotent mesenchymal stromal cells (MSCs) in regenerative and pathological adult neovascularisation [6], [7].

MSCs have typically been characterized by their multi-lineage differentiation potential, giving rise to mesenchymal cell lineages such as adipocytes (fat), osteocytes (bone) and chondrocytes (cartilage) [8]. However, a growing body of literature has uncovered diverse additional functions, including the capacity to promote or modulate angiogenesis by direct interaction with endothelial cells [9], [10]. Cells with MSC properties - often containing a combination of stem cells and more differentiated progeny - have been isolated from a range of tissues including bone marrow, adipose tissue, placenta, skeletal muscle, heart, arterial wall, and endometrium [10], [11]. Due to their frequent identification in vessel walls, and overlapping functional and phenotypic characteristics with pericytes, a perivascular origin of MSCs and a developmental affiliation between the two cell types has been suggested [7], [12]. Furthermore, emerging studies indicate that in addition to driving neovascularisation, hypoxia may also have a role in maintaining MSC stem cell properties [13], [14]. Thus, while their exact origin, phenotype and specific role in neovascularisation remain topics of active debate [4], [7], [15], [16], MSCs have been described as multipotent stromal progenitor cells that are present in the perivascular region of nascent blood vessels and are involved in adult neovascularisation [17]–[20].

Amongst the protein families implicated in regulating vessel patterning, signaling of Eph receptors and their cell-bound ephrin ligands is critical during developmental blood vessel assembly and maturation, but also for sprouting angiogenesis and physiological or pathological adult vessel remodeling [21]–[24]. Ephs are the largest family of receptor tyrosine kinases, comprising (in mammals) nine EphA receptors and five EphB receptors that preferentially interact with six GPI-linked type-A ephrins and three transmembrane type-B ephrins on neighbouring cells [25]. In particular, the role of B-type Ephs and ephrins in guiding endothelial and endothelial/pericyte cell-cell interactions during developmental vascular patterning [21] is well established [26], [27]. Recently, EphB/ephrinB interactions were also shown to control the adhesion and migration of *ex-vivo* expanded MSCs and potentially to be involved in MSC differentiation [28]. On the other hand, the involvement of EphA receptors in adult neovascularisation and tissue repair is poorly understood.

EphA3 functions during embryogenesis in the presomitic mesoderm [29], in stromal and in neuronal tissues [30], [31], and is critical for the endothelial/mesenchymal transition (EndMT) that underlies heart valve development [32]. However, its expression and function in normal adult tissues have not been described. Notably, EphA3 is implicated and recognised as an anti-cancer target in solid and hematopoietic tumors [33], and we recently discovered EphA3 overexpression and function on bone marrow-derived MSCs that are recruited into the vascularised tumour microenvironment [34].

By investigating a potential role of EphA3 during normal adult neovascularisation, we discovered its distinct expression on emerging blood vessels in human endometrium, a tissue lining the uterus that undergoes scheduled cycles of complete regeneration and neovascularisation [35]. Affinity isolation of EphA3^+^ endometrial multipotent mesenchymal stromal cells (eMSCs) from fresh hysterectomy tissue samples and their propagation in culture enabled phenotypic characterization, assessment of clonogenicity and tri-lineage differentiation potential, and assessment of their pro-angiogenic properties *in vivo* by transplantation into immunocompromised mice. Our findings for the first time provide evidence for the hypoxia-controlled expression of EphA3 on human MSCs, and suggest its role in facilitating MSC-supported early stages of regenerative adult neovasculariation.

## Materials and Methods

### Antibodies

The conformation-specific α-EphA3 mouse monoclonal antibody (mAb) IIIA4 [36], and its use for EphA3 activation, immunoprecipitation (IP), immunofluorescence and flow cytometry, as well as in-house-generated anti-EphA3 polyclonal antibodies for Western blots, immunohistochemistry and immunofluorescence analysis, have been described previously [37]–[39]. Non-activating anti-EphA3 mAbs 3D7 (A. Boyd, Queensland Institute of Medical Research) and SL2 (KaloBios Pharmaceuticals), were conjugated to Alexa^647^ and also used to detect EphA3 by flow cytometry and immunofluorescence. The following antibodies were used for immunofluorescence analysis: rabbit α-phosphotyrosine-EphA3 (Millipore/Chemicon), rabbit α-NG2 (Millipore), mouse α-human CD105 (Dako), PDGFR-β (R&D systems), CD49f (clone GOH3, BD) and HIF-1α (clone H1alpha67, Novus Biologicals); CD44-FITC (clone IM7, BioLegend or BD Biosciences), CD90-FITC (clone 5E10, BD), CD73-FITC or PE (clone AD2, BD), CD29-FITC (clone mAb 13, BD), and CD31-Alexa^488^ (clone M89D3, BD). Flow cytometry was done with fluorophore-conjugated mAbs: EphA3 (IIIA4)[38], CD105-V450 (Abcam or BD Biosciences), PDGFR-β-PE (clone PR7212, R&D Systems), CD34-FITC (clone 8G12, BD), KDR/VEGFR-2-PE (clone 89106, R&D systems), CD45-Pacific Blue (clone T22/39, Dako Cytometry), CD90-FITC (clone 5E10, BD), CD73-FITC (clone AD2, BD), CD146-PE (clone P1H12, Miltenyi), CD44-Pacific Blue or FITC (clone IM7, BioLegend or BD); or isotype control antibodies conjugated to FITC, Alexa^488^, PE or Pacific Blue/V450 (BD). Non-conjugated primary antibodies used were against CD106 (clone 51-10C9, BD), CD29 (clone mAb 13, BD) or NG2 (rabbit polyclonal, Millipore). Where required, primary antibody binding was detected by species-specific secondary antibodies conjugated to FITC, Alexa^488^, Alexa^546^ or Alexa^647^ (Life Technologies).

### Immunofluorescence

Cells on fibronectin-coated (10 µg/ml) glass slides (BD Biosciences) were fixed in 4% paraformaldehyde (PFA), permeabilised with 0.2% Triton X-100 and blocked (1 h, room temperature) in phosphate-buffered saline (PBS)/0.2% bovine serum albumin (BSA), prior to incubation (30 min, 37°C) with appropriate antibodies in PBS/0.2% BSA, and Alexa-conjugated secondary antibodies (1 h, room temperature). In some cases cells were stained with fluorescently conjugated antibodies in culture media prior to fixation. Alternatively, actin was stained in permeabilised cells with Alexa^488^-phalloidin (1 µg/ml; Molecular Probes). For cell rounding assays, IIIA4 mAb (1.5 or 3.0 µg/ml), cross-linked with anti-mouse IgG (Jackson ImmunoResearchs), was added to cells 10 min prior to analysis. Coverslips were mounted onto microscope slides with Fluoromount (Sigma). Fluorescence images were taken on a Leica SP5 microscope and analysed using AnalySIS software (Soft Imaging System, Germany).

### Flow cytometry

Cell suspensions in FACS buffer (PBS, 1% fetal calf serum (FCS), 1 mM EDTA) were treated with Fc Receptor blocking reagent (Miltenyi) and labeled (10–15 min, 4°C) with fluorophore-conjugated mAbs. Dead cells were stained with propidium iodide (Sigma) or 7-AAD (Invitrogen), and routine control samples for multivariate flow cytometry included cells incubated with non-relevant isotype-matched control antibodies, unstained cells and Fluorescence Minus One samples. Raw data were analysed and multivariate experiments compensated using FLOWJO software (TreeStar).

### Immunohistochemistry and immunofluorescence of tissue sections

For immunohistochemistry, frozen sections were fixed in acetone, and endogenous peroxidases quenched with 3% H_2_O_2_ prior to incubation with primary antibodies: SL2 α-EphA3 mAb 1 h room temperature; sheep polyclonal α-EphA3 1 h 37°C + overnight, 4°C; mouse α-human CD31(Dako) overnight at 4°C; mouse α-human HIF-1α overnight at 4°C; and appropriate isotype control antibodies. ABC (Vector laboratories) or Novolink (Leica) kits were used for signal amplification, and staining visualized with DAB or AEC chromogens (Vector laboratories). Nuclei were counterstained with Haematoxylin. The specificity of staining with α-EphA3 mAb IIIA4 [38] or sheep polyclonal antibody [39] was assessed by competition with 60-fold molar excess soluble EphA3 exodomain. For immunofluorescence, fluorophore-conjugated primary antibodies were used in some cases (SL2-Alexa^647^, CD31-Alexa^488^). Otherwise, primary antibody binding was detected with Alexa-conjugated secondary antibodies (1 h room temperature). Nuclei were counterstained with DAPI or Hoechst (Sigma).

### Immunoprecipitation

The activity of the anti-EphA3 mAb IIIA4 as agonist has been described previously [38]. For ligand stimulation, ephrinA5-Fc or IIIA4 were pre-clustered using anti-human or anti-mouse IgG (Jackson ImmunoResearch) and cells stimulated for 10 min. EphA3 was immunoprecipitated from whole cell detergent lysates using agarose-conjugated α-EphA3 IIIA4 mAb, and immunoblotted with indicated primary antibodies.

### Endometrial cell isolation

Human endometrial tissues were obtained from women who gave informed written consent for this study, which was approved by the Monash University Human Research and Ethics Committee (CF08/1286 – 2008000625). The tissues were from ovulating women (20–40 years) undergoing hysterectomy without endometrial pathology. Cell suspensions were prepared from endometrial tissues, collected at proliferative and secretory phases of the menstrual cycle, after removal from the underlying myometrium [40] within 12 h after hysterectomy. The tissue, comprising endometrium and 1–2 mm of adjacent myometrium, was finely chopped and digested 2 h at 37°C with 2.5 mg/ml collagenase and 1 mg/ml DNase (Worthington) in PBS, pH 7.4, 0.1% BSA [41]. Single cell suspensions were obtained by filtration (100 µM), and expanded in hypoxic (1–3% O_2_) tissue culture in supplemented Endothelial Basal Growth medium (EBM-Bulletkit, Lonza) containing 20% FCS. These cells are referred to as endometrial stromal cells (eSCs). EphA3^+^eSCs were isolated following non-enzymatic cell detachment (Invitrogen cell dissociation solution) by magnetic activated cell sorting (MACS, MiltenyiBiotec) using Alexa^647^-conjugated IIIA4 or 3D7 α-EphA3 mAbs [38], [42] and α-Alexa^647^ conjugated magnetic beads.

### Quantitative RT-PCR (qRT-PCR)

Samples were analysed by SYBR Green qRT-PCR, with primer pairs listed in Table S1. Relative gene expression was determined using a calibration standard curve [43] and 18S rRNA as reference, or using the Comparative Quantitation method within the Rotorgene 6000 software (v. 6; Corbett Research) with β-actin as Calibrator.

### Gene silencing with shRNA

MISSION Lentiviral Transduction Particles (Sigma) containing control (pLKO.1-puro) or HIF-1α shRNAs (TRCN0000003808, TRCN000000309, TRCN0000003810, TRCN0000003811, TRCN0000010819) were used to transduce EphA3^+^eSCs for 3 days prior to selection with puromycin. EphA3 silencing was done using targeting sequences TRCN0000006408, TRCN0000006409, TRCN00000064010, TRCN00000064018, and clones with maximal silencing after 48 h selected by flow cytometry. Silencing was confirmed in human epithelial kidney (HEK)293T cells with known EphA3 expression [44].

### Colony-forming unit assay

Sorted EphA3^+^ or EphA3^-^ and non-sorted eSC suspensions were seeded into 6-well tissue culture plates at clonal cell density (30 cells/well ≈3 cells/cm^2^) and screened for colonies after adherent culture for a minimum of 21 days in either 20% oxygen, 5% CO_2_ (normoxia), or 1–2% oxygen, 5% CO_2_ (hypoxia).

### Chemotaxis assay

Directed cell migration was analysed in µ-Slide Chemotaxis chambers (ibidi). Cells exposed to a gradient of cross-linked IIIA4 mAb were imaged at 20 min intervals (18 h total) in several randomly selected fields by time-lapse microscopy (AF6000 LX microscope, Leica). Migration co-ordination data for each observed cell was acquired with the ManualTrack plugin in ImageJ software (Fabrice Cordelières, Institut Curie, Orsay, France; http://rsb.info.nih.gov/ij/plugins/track/track.html). Chemotaxis plots and migration velocities of each cell were determined with the Chemotaxis and Migration Tool (ibidi; http://ibidi.com/software/chemotaxis_and_migration_tool).

### 3D *in vitro* co-culture assays

CellTracker Orange (Molecular Probes)-labelled EphA3^+^ or EphA3-depleted eSCs and CellTracker Green-labelled tumour-derived endothelial cells (TECs) were co-cultured at a 1∶2 ratio (6×10^3^ eSCs: 1.2×10^4^ TECs) on growth-factor-reduced Matrigel (BD) in triplicate wells of 15-well µ-Slide Angiogenesis chambers (ibidi). Cells were allowed to interact for>18 h, under stimulation with the following treatments: 5 µg/ml Alexa^647^ human/mouse chimeric (ch)IIIA4 antibody or human Fc as control, cross-linked for 15 min using Alexa^647^ anti-human IgG (Jackson ImmunoResearch); 5 µg/ml EphA3-Fc; or media only (untreated). Cultures were fixed (4% PFA/0.2% glutaraldehyde), permeabilised and nuclei stained with DAPI, followed by widefield 10x tile scan imaging of each well using an AF6000 LX microscope (Leica). The image of each well was then subdivided into quadrants to facilitate batch analysis of images. Cell clustering and segregation were assessed using an adaptation of the ‘Cells’ module in Imaris software (v. 7.5.2, Bitplane) and Imaris Batch Processor (v. 1.1.1), which enabled quantitation of cluster size and number of eSC or TEC per cluster by automated segmentation of DAPI-stained nuclei combined with red or green fluorescence signal thresholds. Statistical analysis was performed using Excel (Microsoft) or Graphpad Prism (v. 6.00, Graphpad). Proportions of cell clusters above a threshold size (>15 eSC nuclei per cluster) were calculated for each quadrant (n ≥ 10) and compared using Student's *t*-test after trimming highest and lowest values. Data regarding number of cells per cluster and cluster area were aggregated from all quadrants and analysed by Kruskall-Wallis test (n ≥ 10 quadrants, typically n>1500 data points per condition).

### MSC multi-lineage differentiation

MACS affinity-purified EphA3^+^eSCs or EphA3-depleted eSCs were differentiated for 3 weeks in StemPro supplemented Osteogenic, Adipogenic or Chondrogenic differentiation media (Life Technologies), or basal media +1% FCS as control, with media refreshed twice weekly. To assess osteogenesis, cells were seeded at 2.5×10^3^ or 5×10^3^ per cm^2^ and stained after 3 weeks for extracellular calcium deposits with 0.1% Alizarin red dye, pH 4.1–4.3. Adipogenesis assays were stained using 0.3% Oil Red dye in 60% isopropanol to detect intracellular neutral lipid deposits. Chondrogenic cellular micromasses were formed by allowing a concentrated drop of cell solution (8×10^4^ in 10 ul) to adhere to a tissue culture well for 2 h before addition of differentiation media. After 3 weeks, pellets were stained overnight using 0.01% Alcian Blue solution in 60% Ethanol/40% Acetic acid.

### *In vivo* angiogenesis

*In vivo* experiments in mice complied with National Health and Medical Research Council (NH&MRC) and Monash University Animal Ethics Guidelines and were approved by the School of Biomedical Sciences and Monash Animal Research Platform Animal Ethics Committees (SOBSB/B/2004/59; MARP/2014/31). All care was taken to minimise pain during the procedures, and animals were euthanized humanely by cervical dislocation at the end of the experiment. Mice were housed in specific pathogen free (SPF) conditions with housing and husbandry according to NH&MRC guidelines.

Ice-cold Matrigel containing EphA3^+^eSCs transduced with EphA3 shRNA or control shRNA-containing lentiviral particles, were injected subcutaneously into the flank of 4-6 week old male Balb/C^nu/nu^ (nude) immunocompromised mice (n ≥ 4 mice per group), as described [45]. Mice were injected intravenously with 100 µg Alexa^594^-IIIA4 mAb 48 h prior and with 1 mg FITC-*Ricinus cummunis* Agglutinin (RCA)-lectin 5 min prior to *in vivo* imaging. Mice were anaesthetised with ketamine (100 mg/kg) and xylazine (15 mg/kg) for imaging, with depth of anaesthesia monitored by assessing respiration rate and responsiveness to toe pinch. The Matrigel transplantation site was then exposed by generating a skin flap, and imaged with a Leica SP5 2-photon microscope (20x Plan Apo 1.0 NA water objective), using external detectors for maximal sensitivity at lowest excitation. 3D vascular volumes from 1 µm-optical sections (512×512 pixels) were estimated using Imaris.

Human EphA3^+^eSCs were imaged in frozen sections of Matrigel plugs with α-human CD73-PE antibodies and Alexa^568^-secondary antibodies. EphA3 was detected using sheep α-EphA3 antibodies, amplified with a biotinylated anti-sheep secondary antibody and Alexa^647^streptavidin. Species-specificity of α-human CD73 was verified on frozen sections of fibrin cell clots with varying proportions of human and mouse cells, using α-mouse CD29-FITC (clone HMβ1-1, eBioscience) and α-FITC Alexa^488^ (Life Technologies) to detect mouse cells. EphA3 expression levels in sections stained by immunohistochemistry were quantitated in thresholded images using Aperio ImageScope (v. 11.2.0.78).

## Results

### EphA3 expression on emerging vasculature

**Figure 1:**
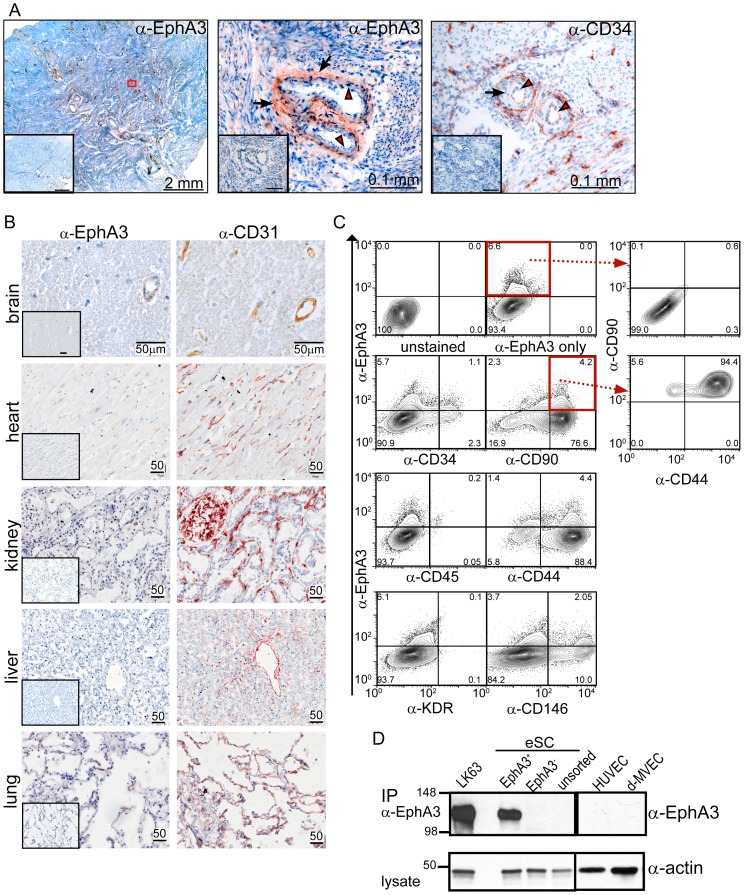
Stromal and perivascular EphA3 expression in regenerating human endometrium. (**A**) Immunohistochemistry of frozen endometrial sections with α-EphA3 and α-CD34 antibodies; the red boxed area in the overview (left) is shown at 40x in the middle panel. Red arrowheads mark CD34^+^ endothelial cells and black arrows EphA3^+^ perivascular cells. Insets show control immunohistochemistry with 10x molar excess recombinant soluble EphA3 (α-EphA3), or secondary antibody only (α-CD34). (**B**) Indicated human tissue sections were analysed using α-EphA3, isotype-matched control (inset), and α-CD31 antibodies to label endothelium; 50 µm scale bars. (**C**) Flow cytometry of endometrial stromal cell (eSC) samples testing co-expression of EphA3 and indicated cell surface markers; fractions of positively stained cells are indicated in each quadrant. Data is representative of 4 independent experiments. (**D**) IP/Western blot analysis of EphA3 expression in unsorted, EphA3^+^ and EphA3-depleted eSCs; LK63 pre-B leukemic cells, HUVECs and dermal microvascular endothelial cells (d-MVEC). Actin was tested to assess sample loading.

To assess a potential implication of EphA3 in physiological adult neovascularisation, we screened tissue sections from a range of vascularised human organs including brain, lung, kidney, liver, and the endometrial layer of the uterus, a tissue where the highly vascularised inner layer (*stratum functionalis*) undergoes monthly cycles of complete regeneration [35]. Hysterectomy samples from the proliferative and early secretory phases of the menstrual cycle revealed EphA3 immunoreactivity mainly in surrounding stroma and on perivascular cell layers of spiral arterioles (Figure 1A, Figure S1), fast-growing blood vessels characteristic for the *stratum functionalis* [35]. EphA3 expression was also detected in the perivascular layers of blood vessels in the *stratum basalis*, the basal layer of the endometrium proposed to contain the stem cell populations responsible for regeneration [46], [47]. The EphA3 staining appeared to vary across the menstrual cycle, from being barely detectable in the early proliferative phase to markedly intense staining in the secretory phase (Figure S1). By contrast, other well-vascularised adult human organs, including brain, heart, liver, kidney and lung showed marginal (brain) or undetectable EphA3 expression (Figure 1B), suggesting its expression particularly in tissues undergoing neovascularisation.

Flow cytometry of endometrial cell suspensions revealed a small population of EphA3^+^, CD34^-^/CD45^-^ cells predominately co-staining for the stromal cell markers CD44, CD90 and CD146 (Figure 1C). This population could be isolated using α-EphA3 antibodies and magnetic beads (MACS; Figure S2 A). Immunofluorescence and qRT-PCR of the resultant plastic-adherent cell preparations from these endometrial samples suggested varying levels of EphA3 expression in different preparations Figure S2 B). IP/Western blot analysis confirmed EphA3 expression in the affinity-purified cells but undetectable levels in the unpurified or EphA3-depleted endometrial stromal cell (eSC) fractions (Figure 1D). EphA3 was also undetectable in various cultured endothelial cell lines, including human umbilical vein vascular endothelial cells (HUVECs), dermal microvascular endothelial cells (d-MVEC), and tumour-derived endothelial cells (TECs) [48]. Having thus isolated a unique population of EphA3^+^eSCs, we subsequently undertook to interrogate the characteristics and properties of these cells with regard to their potential role in regulating neovascularisation.

### Regulation of EphA3 expression by hypoxia inducible factor (HIF)-1α

**Figure 2:**
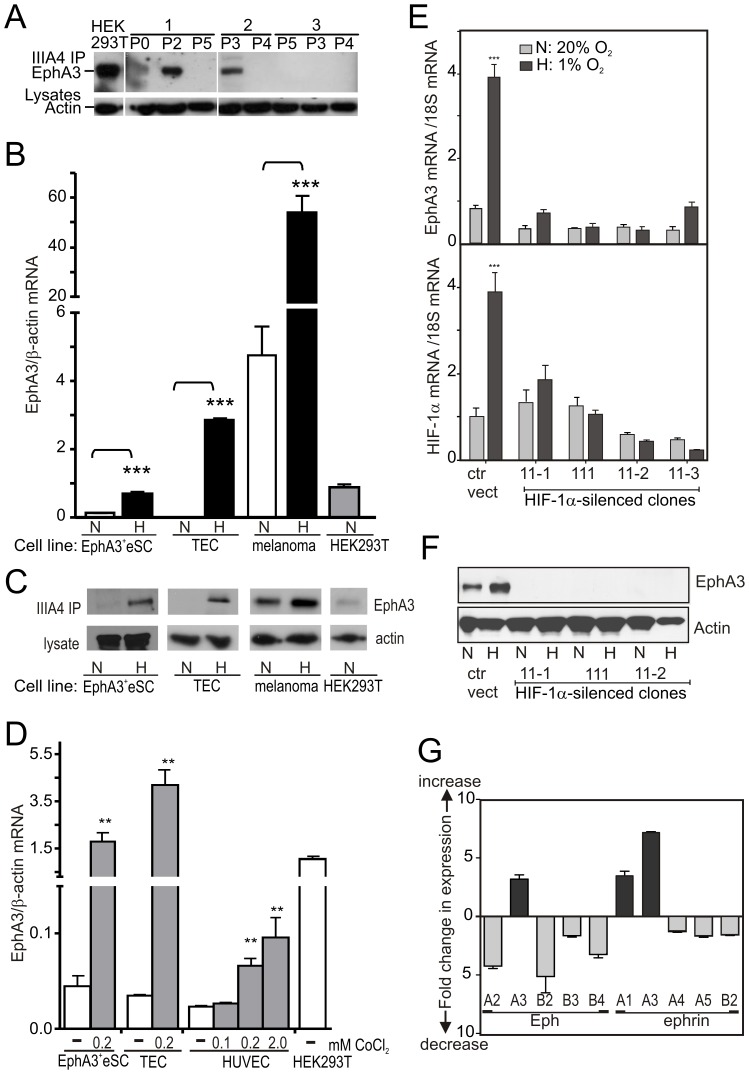
Regulation of EphA3 expression by hypoxia/HIF-1α. (**A**) α-EphA3 IP/Western blot of EphA3^+^ eSC preparations (1-3), after indicated passages (P) in normoxia. HEK293T cells were used as reference for EphA3 expression; actin was used to assess sample loading. (**B**) EphA3 mRNA levels, normalized to β-actin mRNA, were assessed by qRT-PCR in cells cultured (>24 h) in hypoxia (H, ▪) or normoxia (N, □); mean ± SEM (n = 3), ***P<0.005 (Students *t*-test). EphA3^+^eSC, endometrial stromal cells; TEC, tumour-derived endothelial cells; melanoma, AO9 melanoma cells; HEK293T cells used as control. (**C**) IP/Western blot of EphA3 protein in cells cultured in hypoxia or normoxia; 10x more lysate from EphA3^+^eSCs than from HEK293Ts was used. (**D**) CoCl_2_–induced (>24 h) hypoxia increases EphA3 expression: qRT-PCR to assess mRNA levels, normalised to β-actin. (**E**) HIF-1α silencing prevents EphA3 mRNA expression in EphA3^+^eSCs. qRT-PCR of EphA3 (top) and HIF-1α (bottom) mRNA levels in EphA3^+^ eSC clones with shRNA-silenced HIF-1α-expression, cultured in normoxia (▪) or hypoxia (▪). ‘Empty’ pLKO.1 puro vector was used as control (ctrl vect), mRNA levels standardized to 18S rRNA. In (D, E), one way ANOVA was used to test statistical significance, mean ± SEM (n = 3) are shown: ** P<0.01, *** P<0.001. (**F**) EphA3 protein levels in EphA3^+^eSCs. HIF-1α-silenced clones cultured in normoxia or hypoxia were analysed by IP/Western blot. (**G**) Hypoxia-induced changes of Eph and ephrin mRNA expression levels in EphA3^+^eSCs were determined by qRT-PCR. Values are represented relative to the gene with lowest mRNA expression, ephrin-A1, assigned the value 1.0 (see Table S2). Relative change in mRNA levels upon switching from normoxia to hypoxia is shown.

During expansion of the EphA3^+^eSC population in tissue culture, we noticed that repeated passaging under conventional (‘normoxic’, 20% O_2_) conditions resulted in rapid loss of EphA3 expression (Figure 2A). Considering that EphA3 has been identified amongst the hypoxia-regulated genes in bone marrow-derived MSCs [49] and dendritic cells [50], we assessed if oxygen tension would affect EphA3 expression. Hypoxic (1–2% O_2_) culture of EphA3^+^eSCs, as well as cells with low/undetectable EphA3 (TECs) or with measurable EphA3 expression (A09 melanoma [37]), significantly increased EphA3 mRNA levels in all cases (Figure 2B, C). Likewise, exposure to CoCl_2_-induced hypoxia increased EphA3 mRNA expression in EphA3^+^eSCs and TECs 30-60-fold (Figure 2D), and moderately in HUVECs.

To assess if the key hypoxia response gene HIF-1α regulates EphA3 expression, we examined mRNA and protein levels in EphA3^+^eSC clones after shRNA-mediated HIF-1α silencing. Indeed, HIF-1α silencing (Figure 2E, bottom) in all clones resulted in concomitant loss of EphA3 mRNA (Figure 2E, top) and protein expression (Figure 2F). Additionally, immunostaining indicated broad expression of HIF-1α across secretory phase endometrium, including in some EphA3^+^ perivascular cells (Figure S3), suggesting that HIF-1α likely regulates EphA3 expression *in vivo*.

While compared to other Ephs and ephrins the normoxic EphA3 expression in the isolated EphA3^+^eSCs was moderate (Table S2), we found that hypoxia significantly enhanced expression of EphA3 and its ligands ephrin-A1 and ephrin-A3, while levels of other Ephs and ephrins with established roles in neovascularisation were notably attenuated (Figure 2G). Together our analysis reveals that EphA3 expression in primary EphA3^+^ eSCs and in established cell lines increases under hypoxic conditions, whereas the expression of other Ephs and ephrins in EphA3^+^eSCs is notably reduced.

### EphA3 marks a population of endometrial MSCs

**Figure 3:**
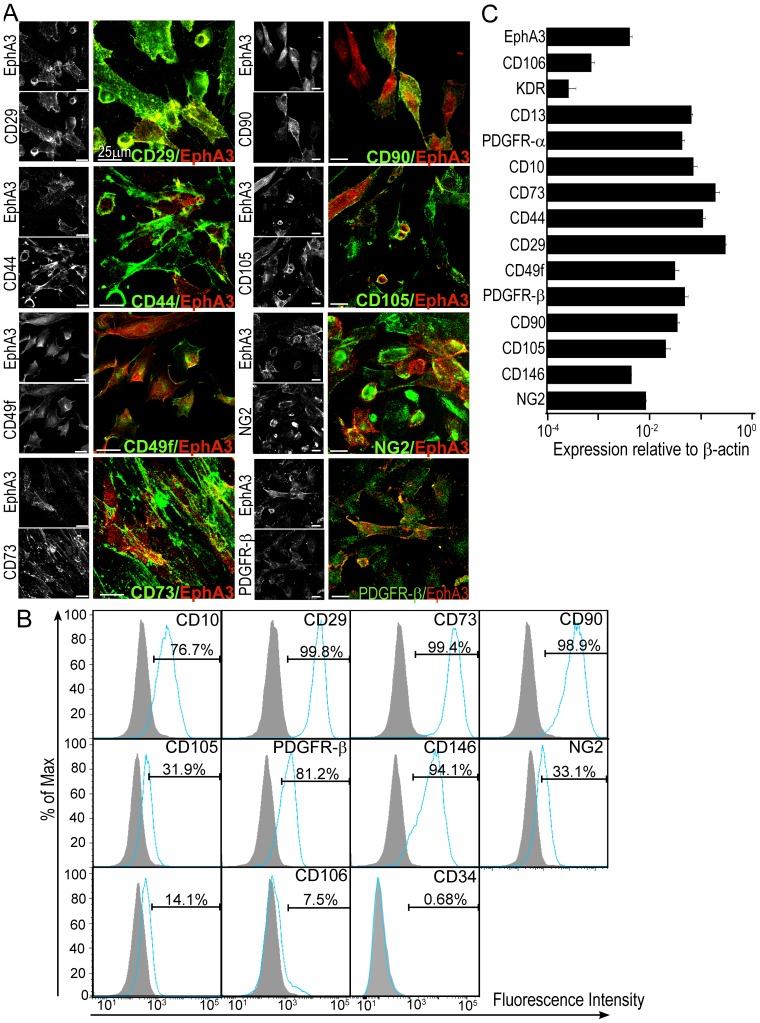
Phenotypic characterization of EphA3^+^ endometrial stromal cells. (**A**) Immunofluorescence analysis of EphA3^+^eSCs using antibodies to EphA3 (red) and indicated antigens (green), characteristic of an MSC cell surface expression profile. Individual fluorescent channels and merged images are shown, 25 µm scale bars. (**B**) Cell surface expression profile of indicated antigens on EphA3^+^eSCs was assessed by flow cytometry; filled peaks represent isotype-matched controls. (**C**) The mRNA expression levels of indicated genes (relative to β-actin) were assessed in EphA3^+^eSCs by qRT-PCR. Mean and standard deviation (n = 3) are shown.

Considering that our observation of perivascular EphA3 expression in endometrium corresponds to the commonly-observed location of MSCs in various tissues [12], and that hypoxia helps to maintain MSC properties [13], [14], we explored whether EphA3^+^eSCs might possess the phenotypic and functional characteristics of MSCs. Indeed, confocal microscopy, flow cytometry and qRT-PCR of EphA3^+^eSCs revealed a cell surface expression profile that is suggested to be characteristic for MSCs [6], [7], [12], [51], including expression of CD10, CD13, CD29, CD73, CD90, CD105, CD146, CD49f/integrin-α6, platelet-derived growth factor receptor (PDGFR)-α, as well as PDGFR-β and NG2 which are also characteristic of pericytes [11] (Figure 3A–C). Conversely, cell surface markers typical of endothelial cells, such as CD34, KDR/vascular endothelial growth factor (VEGF) receptor-2 and CD106/vascular cell adhesion molecule-1 were barely expressed, confirming the perivascular/mesenchymal phenotype of these cells.

**Figure 4:**
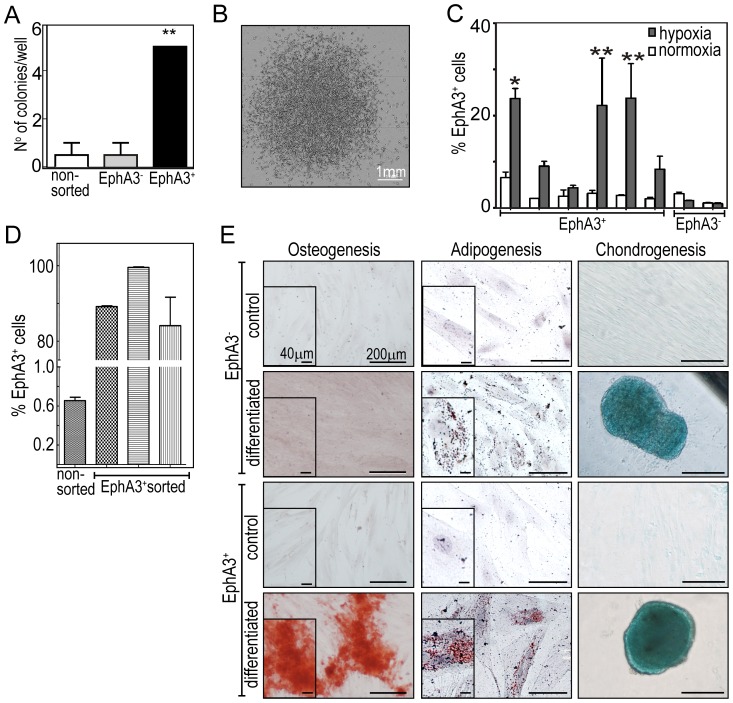
Colony formation and mesenchymal tri-lineage differentiation properties of EphA3^+^ endometrial stromal cells. (**A**) Colony forming potential was scored in non-sorted (□), EphA3-depleted (▪) and EphA3^+^ (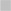) eSCs 2-3 weeks after seeding at clonal density (3 cells/cm^2^) in 6-well tissue culture plates. Mean and SEM are shown, ** P<0.01 (paired Students *t-*test). (**B**) Representative phase-contrast micrograph of a typical EphA3^+^eSC colony on day 21. (**C**) EphA3^+^ and EphA3^-^ eSC colonies were cultured in normoxia (□) or hypoxia (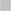) and derived single cell suspensions analysed for EphA3 by flow cytometry: mean ± SEM of total EphA3^+^ cells (n = 3) are shown. * P<0.05 ** P<0.01 (two-way ANOVA). (**D**) EphA3^+^eSCs, FACS-isolated from 3 clones with highest EphA3 expression in (C), and cultured in hypoxia for 3 passages, were re-analysed for EphA3 expression. Non-sorted eSCs illustrate baseline EphA3 expression. (**E**) *In vitro* multi-lineage differentiation of MACS affinity-purified EphA3^+^eSCs was compared to EphA3-depleted eSCs. Cells were grown for ≥3 weeks in control or supplemented osteogenic, adipogenic or chondrogenic differentiation culture media, and stained for osteoblast (alizarin red), adipocyte (oil red O) or chondrocyte (alcian blue) determinants. Representative images are shown from n≥5 experiments. Insets illustrate images at increased magnification; 200 µm and 40 µm (insets) scale bars.

We therefore explored if these cells also displayed the clonogenic and tri-lineage differentiation potential of MSCs [8], [51], [52]. Propagation of fluorescence-activated cell sorting (FACS)-purified EphA3^+^eSCs at clonal cell density revealed their significantly higher capacity for colony formation (16.7%), as compared to EphA3-depleted or unsorted eSCs (3.3%; Figure 4A, B). Notably, we found that hypoxic tissue culture for 10 passages retained EphA3 expression on some 25% of cells in 3 out of 6 EphA3^+^eSC clones (Figure 4C), and that after additional FACS purification EphA3 was maintained on 90–100% of cells for at least two passages (Figure 4D). By contrast, normoxic cell culture decreased the number of EphA3-positive cells in all clones to similar levels (Figure 4C).

In agreement with the tri-lineage differentiation capacity typical for MSCs [8], [51], [52], MACS affinity-purified EphA3^+^eSCs showed osteogenic, chondrogenic and adipogenic differentiation capacity *in vitro* (Figure 4E, bottom panels). Conversely, the EphA3-depleted eSC fraction showed reduced capacity for adipocyte differentiation and predominately failed to form osteoblasts (Figure 4E, top panels). Together, these findings indicate that EphA3^+^ endometrial stromal cells have the properties of MSCs; we thus designated them as EphA3^+^eMSCs (endometrial multipotent mesenchymal stromal cells).

### EphA3^+^eMSCs respond to EphA3 agonists by activation, cell-cell segregation and repulsion

**Figure 5:**
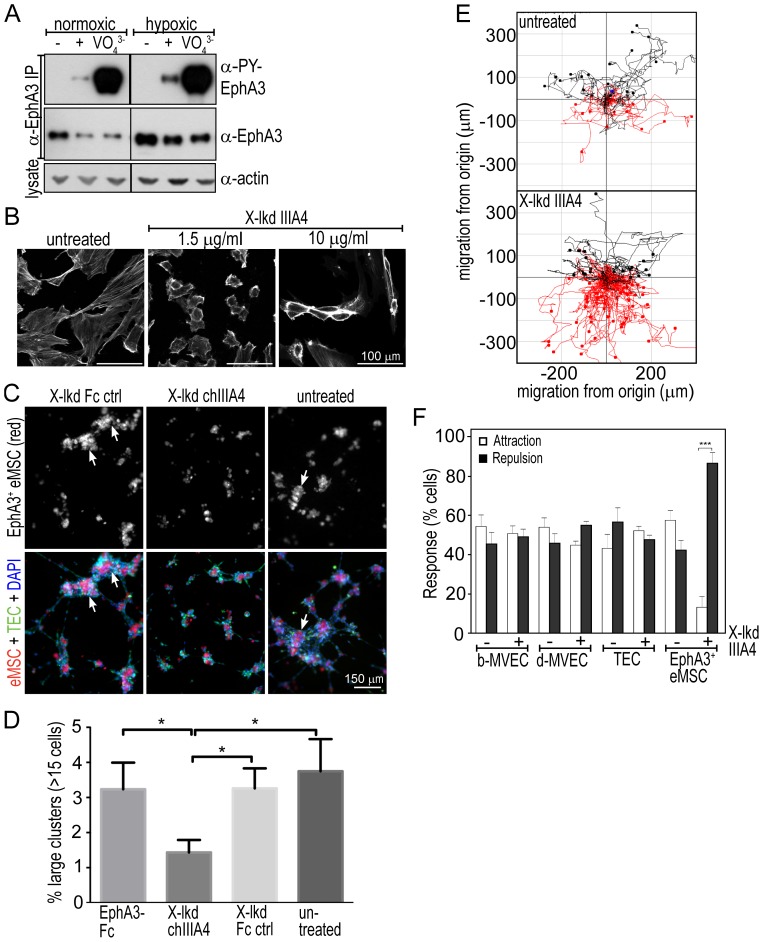
EphA3 activation triggers phosphorylation, cell contraction, cell repulsion and cell-cell segregation of EphA3^+^eMSCs. (**A**) Western blots of α-EphA3 immunoprecipitates from non-stimulated (-), pre-clustered ephrinA5-Fc stimulated (+), or sodium-vanadate (VO_4_^3-^) treated EphA3^+^eMSCs grown in normoxia or hypoxia. Actin was used to assess protein loading. (**B**) Confocal images (Alexa^488^-phalloidin, cytoskeleton) of EphA3^+^eMSCs stimulated with crosslinked (X-lkd) IIIA4 mAb; scale bar: 100 µm. (**C**) Organoids formed by EphA3^+^ eMSC (red) and TEC (green) 3D co-cultures in Matrigel; nuclei stained with DAPI (blue). Treatments (>18 h) were: cross-linked (X-lkd) chimeric (ch)IIIA4 mAb [88], crosslinked control Fc protein (Fc ctr), EphA3-Fc, or untreated. Representative fields of the red and merged channels are shown. Arrows indicate clusters>15 cells as quantitated in (D). (**D**) Individual cells within clusters were defined and quantified through software-based automatic segmentation of DAPI^+^ nuclei; the mean (+/− SD) fraction of large clusters (>15 nucleated cells/cluster) forming under the different treatments (n≥10 quadrants) are illustrated; * p<0.05 (Students *t*-test). (**E**) EphA3-directed cell migration (repulsion) of EphA3^+^eMSCs, exposed to a gradient of cross-linked IIIA4 mAb (bottom panel) or left untreated (top panel). The path of individual cells (n≥30) in each field of view (n≥4) is plotted: cells in the lower half of each diagram have moved away (red, repulsion), those in the upper moved towards (black, attraction) the line of reference. (**F**) Directed migration of indicated cells in the presence (+) and absence (−) of a gradient of cross-linked IIIA4 mAb is quantitated as cell attraction (□) or cell repulsion (▪). Mean ± SEM are shown (n = 4), *** P<0.001 (two-way ANOVA).

We next evaluated functional responses of the EphA3^+^eMSCs to EphA3 agonists. *In vitro* activation of Ephs is achieved with clustered ephrin-Fc fusion proteins [53], so we treated cells with pre-clustered ephrin-A5-Fc, the preferred ligand for EphA3 [54]. While EphA3 was not phosphorylated in cultured eMSCs, pre-clustered ephrin-A5-Fc elicited robust phosphorylation in normoxic and hypoxic cells, which indicates the activation of kinase-dependent EphA3 signalling [53] (Figure 5A). EphA3 phosphorylation levels were significantly higher after treatment with the pan-specific protein tyrosine phosphatase inhibitor sodium-pervanadate, suggesting that EphA3 kinase activity in these cells may be tightly controlled by protein tyrosine phosphatases [55]. Treatment with cross-linked α-EphA3 mAb IIIA4, previously shown to specifically activate only EphA3 [38], triggered notable cell contraction (Figure 5B), previously shown to be a default response to EphA3 activation [37], [38], [55].

We thus tested responses to EphA3 activation in an experimental three-dimensional (3D) cell-cell interaction model. In Matrigel, co-cultures of tumour-derived endothelial cells and eSCs assembled into organoids where endothelial cells wrapped around a central mesenchymal/stromal cell cluster, as was observed in HUVEC/MSC co-cultures in another study [23] (Figure S4 A). Interestingly, we noted a significantly higher frequency of larger organoids forming from EphA3^+^eMSCs as compared to EphA3-depleted eSCs (Figure S4 B), whereby EphA3^+^eMSC clustering occurred independently of the presence of endothelial cells (Figure S4 C). Importantly, the frequency of larger organoids was significantly reduced when the two cell types were co-cultured in the presence of cross-linked chIIIA4 antibody [38] (Figure 5C, D), suggesting that while EphA3 expression in eMSC promoted homotypic cell-cell interaction and hence the formation of larger organoids, this relied on dormant EphA3 kinase activity.

We next assessed in a cell migration assay if EphA3^+^eMSCs would be repelled by a stationary gradient of an EphA3 agonist. Indeed, a gradient of cross-linked IIIA4 specifically directed the migration of EphA3^+^eMSCs away from the region of highest agonist concentration (Figure 5E, F), whereas none of the other tested cell lines responded to the gradient. Together with our finding from the 3D co-culture model, this is suggestive of a role for kinase-dependent EphA3 activity in guiding the positioning of MSCs, while kinase-dormant EphA3 binding may subsequently stabilise intercellular junctions during endometrial blood vessel assembly.

### EphA3^+^eMSCs contribute to adult neovascularisation

**Figure 6:**
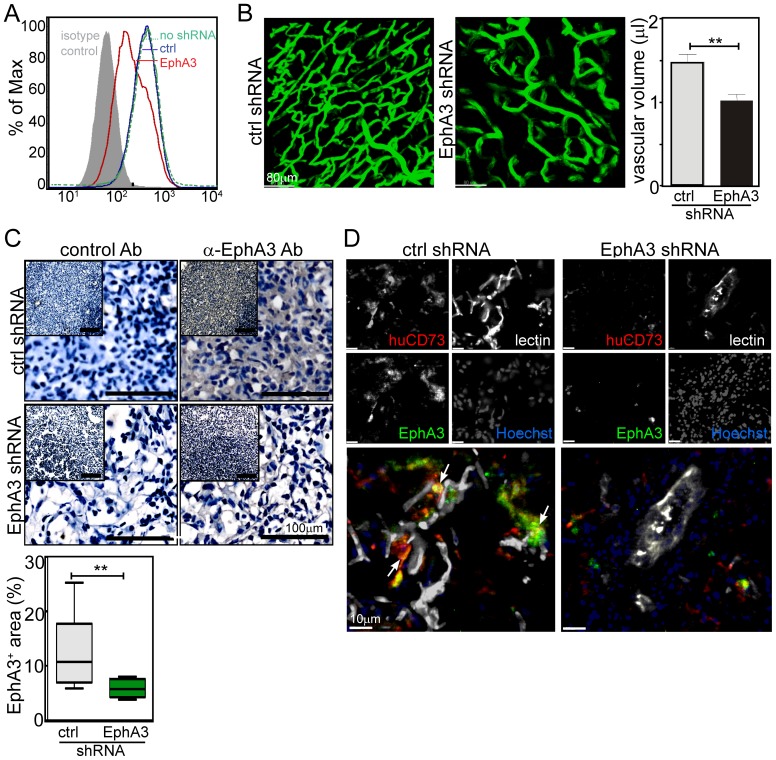
EphA3^+^ eMSCs contribute to blood vessel assembly *in vivo*. (**A**) Validation of reduced EphA3 cell surface expression on HEK293T cells after transduction with EphA3-specific or control shRNA lentiviral particles by flow cytometry. (**B**) 2-photon images of Matrigel plugs containing lentiviral shRNA-transduced EphA3^+^eMSCs 19 days post transplantation, with blood vessels delineated by injected [FITC]RCA-lectin (green); typical fields of view (n ≥ 4 mice) are shown, scale bar 80 µm. The volume of lectin-stained microvessels was estimated from 3D images (n ≥ 7/mouse, ≥ 4 mice/group), ** p<0.01, unpaired Student's *t*-test. (**C**) EphA3 expression in resected Matrigel plugs analysed with α-EphA3 antibodies or secondary α-sheep antibodies only (control Ab). Representative sections (insets) are shown at 4-fold magnification, 100 µm scale bars. α-EphA3-stained areas in ≥3 sections/Matrigel plug/mouse (≥ 3 mice/group): mean, 25% and 75% percentile, minimum and maximum for each group are shown, ** p = 0.004 (2-tailed Student's *t*-test). (**D**) EphA3^+^ (sheep α-EphA3, green) transplanted human eMSCs (α-human-CD73, red) and blood-perfused vessels (RCA-lectin, white) were imaged in resected Matrigel plugs; nuclei were counterstained with Hoechst (blue). Arrows indicate points of close interaction between EphA3^+^/huCD73^+^eMSCs and lectin-stained blood vessels. Individual channels and merged images are shown, scale bars, 10 µm.

To evaluate this notion under more physiological conditions, we explored potential pro-angiogenic properties of EphA3^+^eMSCs *in vivo* by subcutaneous injection as 3D Matrigel cultures into immunocompromised (nude) mice. Prior to intravital microscopy mice were injected with Alexa^594^-IIIA4 mAb and [FITC]RCA-lectin, to label EphA3-positive cells and blood-perfused vessels, respectively. 2-photon imaging of the exposed engraftment sites after 5 weeks revealed a blood-perfused (i.e., lectin-stained) vascular network, in which some of the blood vessels and the surrounding stroma were labeled with Alexa^594^IIIA4, while the adjacent microvessels of the normal mouse skin were devoid of IIIA4 staining (Figure S5). We thus used this local transplantation model to assess a potential function of EphA3 by silencing its expression in engrafted EphA3^+^eMSCs using lentiviral-expressed shRNA (silencing was validated in HEK293T cells; Figure 6A). Imaging of engraftment sites containing EphA3-silenced EphA3^+^eMSCs revealed significantly reduced neovascularisation as compared to control shRNA-transduced EphA3^+^eMSCs (Figure 6B). As expected, reduced vascularity coincided with a significantly reduced expression of EphA3 within the Matrigel transplantation sites (Figure 6C). Immunofluorescence analysis of these tissues using α-EphA3 and human-specific α-CD73 antibodies (Figure S6) indicated the persistence of eMSCs in the Matrigel plugs (Figure 6D). The eMSCs were preferentially located in close proximity to RCA-lectin-stained vascular structures, though this pattern was markedly less evident in Matrigel plugs containing EphA3-silenced eMSCs (Figure 6D). This *in vivo* data further suggests that EphA3 expression in MSCs plays a role in supporting neovascularization in adult tissues.

## Discussion

During mammalian tissue growth and maintenance hypoxia inducible transcription factors respond to oxygen depletion by activating vasculogenic/angiogenic gene programs that orchestrate the assembly of new blood vessels [1]. Apart from pro-angiogenic growth factors, neovascularisation relies on Eph and ephrin cell surface proteins that guide endothelial and mural cell positioning and cell-cell interactions [56]. We now demonstrate for the first time HIF-1α-induced expression of EphA3 on perivascular human MSCs isolated from endometrial tissue, and provide evidence suggesting its role in promoting adult neovascularisation.

### Hypoxia-regulated expression of EphA3 in neovascularisation

There is a growing body of evidence that MSCs from many diverse tissue types can support the growth and stabilization of nascent blood vessels [6], [10]. Our finding of prominent EphA3 expression in endometrial spiral arterioles and surrounding stroma, but not in other human tissues, suggests EphA3 as a unique marker of perivascular MSCs that are implicated in rapid neovascularisation and vascular remodeling. We have also observed this selective EphA3 expression in actively growing rather than established blood vessels in the vascular microenvironment of solid tumours [34]. In this way, the expression pattern of EphA3 contrasts with other Eph and ephrin family members such as EphA2, EphB2, EphB4 and ephrin-B2, which are regarded as endothelial cell surface markers of stable, established blood vessels and endothelial cell lines [26], [27]. Our *in vitro* analyses suggested that kinase-active EphA3 signalling – resulting in cell rounding and increased motility – may be involved in the initial migration and recruitment of EphA3^+^MSCs into nascent blood vessels, while kinase-inactive EphA3-ligand interactions may later help to stabilise cell-cell interactions in the perivascular layer.

As in many other biological contexts, including tumourigenesis, regeneration and revascularisation in the endometrium is critically regulated by hypoxia [1], [57]. Interestingly, we found that while short-term normoxic (20% O_2_) culture of EphA3^+^eMSCs resulted in loss of detectable EphA3, hypoxic culture stimulated expression in these and other cell lines. HIF-1α is strongly implicated in this upregulation, since its silencing blocks EphA3 expression. The transcriptional effect of HIF-1α on EphA3 may not be direct, however, given that the EphA3 promoter appears to lack a hypoxia-response element (HRE), the key motif for HIF-induced transcriptional activation [50]. In support of this *in vitro* data, we detected HIF-1α in perivascular EphA3^+^eMSCs of secretory phase endometrial tissue sections, as well as in some endothelial cells and other cell types. Notably, HIF-1α expression is highest in the secretory phase of the menstrual cycle [58], which corresponds with the highest EphA3 expression observed in our study. HIF-1α is known to induce expression of the key pro-angiogenic growth factor VEGF-A in endometrial stromal and epithelial cells, and while both HIF-1α and VEGF-A are expressed in a range of endometrial cell types, VEGF-A expression has been detected in endometrial perivascular cells [59]–[61]. Thus, the pro-angiogenic function of EphA3^+^eMSCs, subsequent to their EphA3-directed recruitment and integration into the vessel, may include paracrine stimulation of endothelial cells by HIF-1α-induced VEGF-A expression.

Interestingly, our in vitro studies revealed that in addition to EphA3, also the expression of two of its ligands, ephrin-A1 and -A3, was up-regulated 3-6-fold in hypoxia, while levels of EphA2, B2, B3, B4 and ephrin-A4, -A5, and -B2 were all notably attenuated. This hypoxia-induced downregulation seems to contrast an earlier study, which reported increased expression of EphA2, EphB4, ephrin-B2 and ephrin-A1 in whole samples of mouse skin after experimental hypoxia [62]. The apparent discrepancy between our findings and this study may be due to differences in the experimental conditions, but potentially also to species-specific (mouse *versus* human) or tissue- or cell-type-specific differences in the regulation of Ephs and ephrins. Nonetheless, enhanced EphA3 expression during hypoxia agrees with the notion of its expression during recruitment and integration of pro-angiogenic MSCs into nascent blood vessels, which ceases once blood flow results in oxygenation, whereupon other Eph family members are expressed to facilitate cell-cell interactions during blood vessel maturation [26].

In this context, our demonstration of the pro-angiogenic activity of EphA3^+^eMSCs *in vivo* and of EphA3-directed endometrial MSC migration and endothelial/MSC interaction *in vitro* strongly supports the notion that EphA3 functions in human MSCs as a cell guidance receptor that is implicated in the early stages of adult blood vessel formation.

### EphA3 marks a population of multipotent mesenchymal stromal cells

There is now compelling evidence that locally-recruited [63] and bone marrow-derived progenitor cells [64]–[68] are involved in revascularisation of the human endometrium. Recent studies have investigated the regenerative and pro-angiogenic therapeutic potential of eMSCs isolated using various combined or single cell surface markers [69], [70]. Indeed, endometrial-derived MSC-like cells isolated from menstrual blood - also termed “Endometrial Regenerative Cells” - that have multi-lineage differentiation capacity are being evaluated in ongoing Phase-II clinical trials for their cardiovascular regenerative potential [71]. The therapeutic function of eMSCs and related cells is likely multifaceted, including promotion of vascularisation, secretion of trophic factors, and direct differentiation into relevant cell types [70]–[76]. Our present study has identified EphA3 as a new marker of multipotent MSCs in the endometrium, which furthermore is functionally implicated in hypoxia-induced rapid neovascularisation.

Several recent studies suggest that MSCs, termed ‘vascular stem cells’ by some authors [6], [7], [20], can participate in blood vessel formation by recruitment into nascent blood vessels where they can differentiate into vascular and stromal cell types [10], [20], [77], [78]. Furthermore, others have demonstrated vascular and stromal differentiation capacity of MSCs isolated from human endometrium [79], [80], and the underlying plasticity between MSCs and endothelial cells was suggested from studies indicating that TGF-β or BMP4-induced EndMT can convert adult endothelial cells into multipotent MSCs [81]. Of note, EphA3 is essential for EndMT during cardiovascular development, where EphA3^-/-^ mice develop hypoplastic heart valves due to a failure of endothelial cells to undergo transdifferentiation into mesenchymal endocardial cushion cells [32]. In this light, the expression of EphA3 in growing endometrial vessels and adjacent stroma, together with their demonstrated multipotency, hints that EphA3^+^eMSCs *in situ* may give rise to multiple vascular and mesenchymal/stromal cell types.

Importantly, a number of recent studies have revealed that the stem cell properties of MSCs in general are strictly controlled by hypoxia, due to HIF-1α-induced downregulation of E2A/p21 and maintenance of Oct4 and Nanog expression [13], [14]. Thus, normoxic culture or Oct4 and Nanog silencing results in loss of multi-lineage differentiation- and long-term proliferation potential of bone marrow-derived MSCs [13], [14], while hypoxic culture was reported to enhance the overall therapeutic capacity of MSCs [82]–[85]. Meanwhile, another study identified that hypoxia upregulated EphA3, among other genes, in bone marrow-derived MSCs [49]. Considering the substantive correlation between EphA3 expression, hypoxic tissue culture and clonogenic/multilineage differentiation potential of MSCs, it thus is plausible that EphA3 expression may mark a less-differentiated MSC type. Indeed, this correlation warrants further investigation to better understand the function of EphA3 in MSCs during tumorigenesis and tissue regeneration.

## Conclusions

In conclusion, our study for the first time provides detailed evidence for hypoxia-controlled EphA3 expression and function on a human multipotent mesenchymal stromal cell population that participates in the assembly of emerging or regenerating adult blood vessels. While further functional characterization of EphA3 in MSCs during adult neovascularisation is ongoing, our parallel studies in solid tumour progression indicate notable EphA3 expression and function also in the vascularised tumour microenvironment of solid and haematopoietic tumours [33], [34]. Its successful targeting with our therapeutic anti-EphA3 mAb, KB004, which showed promising clinical responses in Phase-I clinical trials (NCT01211691 [86]) including particularly stromal normalisation in patients with fibrotic disease [87], suggest EphA3 as an early marker of adult neovascularisation and as a molecular target for therapeutic applications.

## Supporting Information

Figure S1 **EphA3 IHC of tissue sections from secretory and proliferative phase endometrium.** Fresh-frozen endometrial tissue sections at various phases in the menstrual cycle were incubated with α-EphA3 antibodies followed by secondary antibodies and detection using AEC chromogen. A control section of a late-proliferative sample was treated with secondary antibody only; scale bar, 100 µm (left column), sections in the right-hand column are at higher magnification, scale bar, 50 µm.
(TIF)

Figure S2 **EphA3 expression in human endometrial stromal cells.** (**A**) Flow cytometry of endometrial stromal cells (eSCs) fractionated by MACS into EphA3^+^ (example shown after two rounds of isolation) and EphA3-depleted (EphA3^-^) eSCs. LK63 pre-B leukemic cells serve as a positive control for binding of the IIIA4 α-EphA3 antibody. Shaded peak indicates isotype control-stained cells. (**B**) qRT-PCR of several different EphA3^+^eSC preparations (a–d) at passage 3, relative to β-actin expression. HEK293 cells were used as a control cell line with established EphA3 expression. (**C**) Fluorescence microscopy of MACS-isolated EphA3^+^ eSCs from different preparations; PFA-fixed and permeabilised cells were stained with rabbit α-EphA3 antibodies, Alexa^488^-conjugated secondary antibodies and Hoechst nuclear stain. Fluorescent (2^nd^ Ab only) and phase contrast (phase) micrographs of cells stained with Alexa^488^-labelled secondary antibodies are shown as controls, scale bars: 40 µm.
(TIF)

Figure S3 **Immunofluorescence detection of HIF-1α in human endometrium.** Frozen sections of secretory-phase human endometrium were immunostained for EphA3 (red) and HIF-1α (green), along with CD31 antibodies to mark endothelial cells (white) and Hoechst to stain nuclei (blue). Boxed sections are shown magnified 2x in the panels to the right. Arrows indicate EphA3/HIF-1α co-staining in perivascular cells. Results are representative of n = 6 independent samples. Examples shown are: (A) a large vessel in the basal layer; (B) smaller spiral arterioles in the functional layer; (C) secondary antibodies only as negative control. Scale bar: 30 µm.
(TIF)

Figure S4 **EphA3^+^eMSCs promote the assembly of MSC/endothelial cell organoids.** (**A**) The assembly of 3D cell clusters from EphA3^+^eMSC (red) and tumour endothelial cells (TECs) or human microvascular endothelial cells (HMEC; green) at indicated cell ratios was analysed in overnight co-cultures in growth-factor-reduced Matrigel. Independent of cellular ratios, TECs and HMECs interact with eSCs by forming an outer cell layer around a central eSC cluster. (**B**) 3D eSC/endothelial cell clusters from 1∶2 ratios of EphA3^+^eMSC (EphA3^+^) or EphA3-depleted (EphA3^-^) eSC and TECs. While TECs interacted with both stromal cell populations, EphA3^+^eMSCs revealed significantly increased frequency of forming larger organoids. Mean and SE are shown, * p<0.05 (Student's *t*-test); **** p>0.0001 (Kruskall-Wallis test). (**C**) Assembly into large cell clusters is independent of endothelial cells. Images depict overnight cultures of EphA3^+^ eMSCs and EphA3-depleted eSC (EphA3^-^) alone on growth-factor-reduced Matrigel. Mean and SE are shown; *** p = 0.0003 (Kruskall-Wallis test). Representative fields of views are illustrated in all images (A–C), all indicated scale bars are 100 µm.
(TIF)

Figure S5 **Localisation of fluorescently-labelled α-EphA3 antibodies to growing blood vessels** ***in vivo*** **.** Intravital 2-photon microscopy of subcutaneous Matrigel plugs with EphA3^+^eMSCs, 5 weeks after implantation. Mice had been injected intravenously with Alexa^594^IIIA4 to detect EphA3 expression (red) and [FITC]RCA-lectin to delineate blood vessels (green). Normal skin sections adjacent to the Matrigel (bottom panels) were imaged as controls. Individual and merged fluorescent channels are shown; 80 µm scale bars. Arrowheads indicate EphA3^+^ vessels; stars indicate IIIA4-stained perivascular stromal tissue.
(TIF)

Figure S6 **Species-specific detection of human eSCs.** The species specificity of the α-human CD73 antibody used to detect transplanted EphA3^+^eMSCs in mice was validated using frozen sections of fibrin cell clots. Clots composed of 100% unsorted human (hu) eSCs, 100% mouse embryonic stem cells (mESCs), or 90% mESC/10% human eSC were sectioned and immunostained with a combination of α-human CD73 (red) and α-mouse CD29 (green) antibodies. Staining of human or mouse cells with these antibodies is non-overlapping. Scale bar: 40 µm.
(TIF)

Table S1 **qRT-PCR primer sequences.**
(DOCX)

Table S2 **mRNA expression profile of Ephs and ephrins in EphA3^+^eSCs.** The mRNA expression levels of indicated Ephs and ephrins (epn) in EphA3^+^eSCs, cultured under ^γ^normoxic (20% O_2_) or ^‡^hypoxic (1% O_2_) conditions, were determined by qRT-PCR. ^*^The gene with the lowest mRNA expression is designated as calibrator (ephrinA1) with the value 1.0. ^§^Fold relative changes of mRNA levels under hypoxic versus normoxic conditions for the indicated Ephs and ephrins are shown.
(DOCX)
