# Supplementary figures and images for: Hypoxia-Controlled EphA3 Marks a Human Endometrium-Derived Multipotent Mesenchymal Stromal Cell that Supports Vascular Growth
Source: PLoS One. 2014 Nov 24;9(11):e112106. doi: 10.1371/journal.pone.0112106 (PMC4242616; doi:10.1371/journal.pone.0112106)

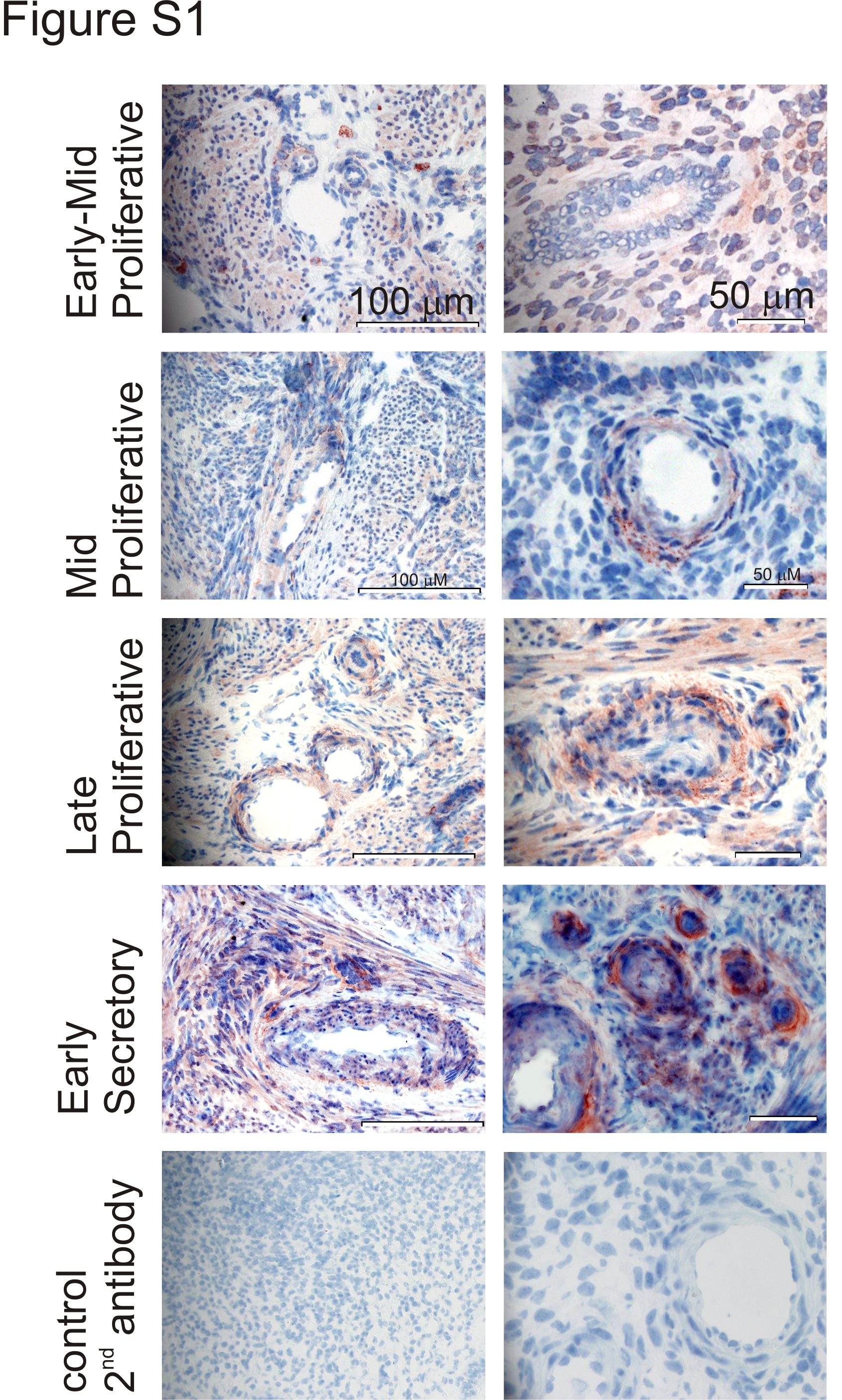

Supplement: Figure S1 — EphA3 IHC of tissue sections from secretory and proliferative phase endometrium. Fresh-frozen endometrial tissue sections at various phases in the menstrual cycle were incubated with α-EphA3 antibodies followed by secondary antibodies and detection using AEC chromogen. A control section of a late-proliferative sample was treated with secondary antibody only; scale bar, 100 µm (left column), sections in the right-hand column are at higher magnification, scale bar, 50 µm. (TIF) [file pone.0112106.s001.tif]

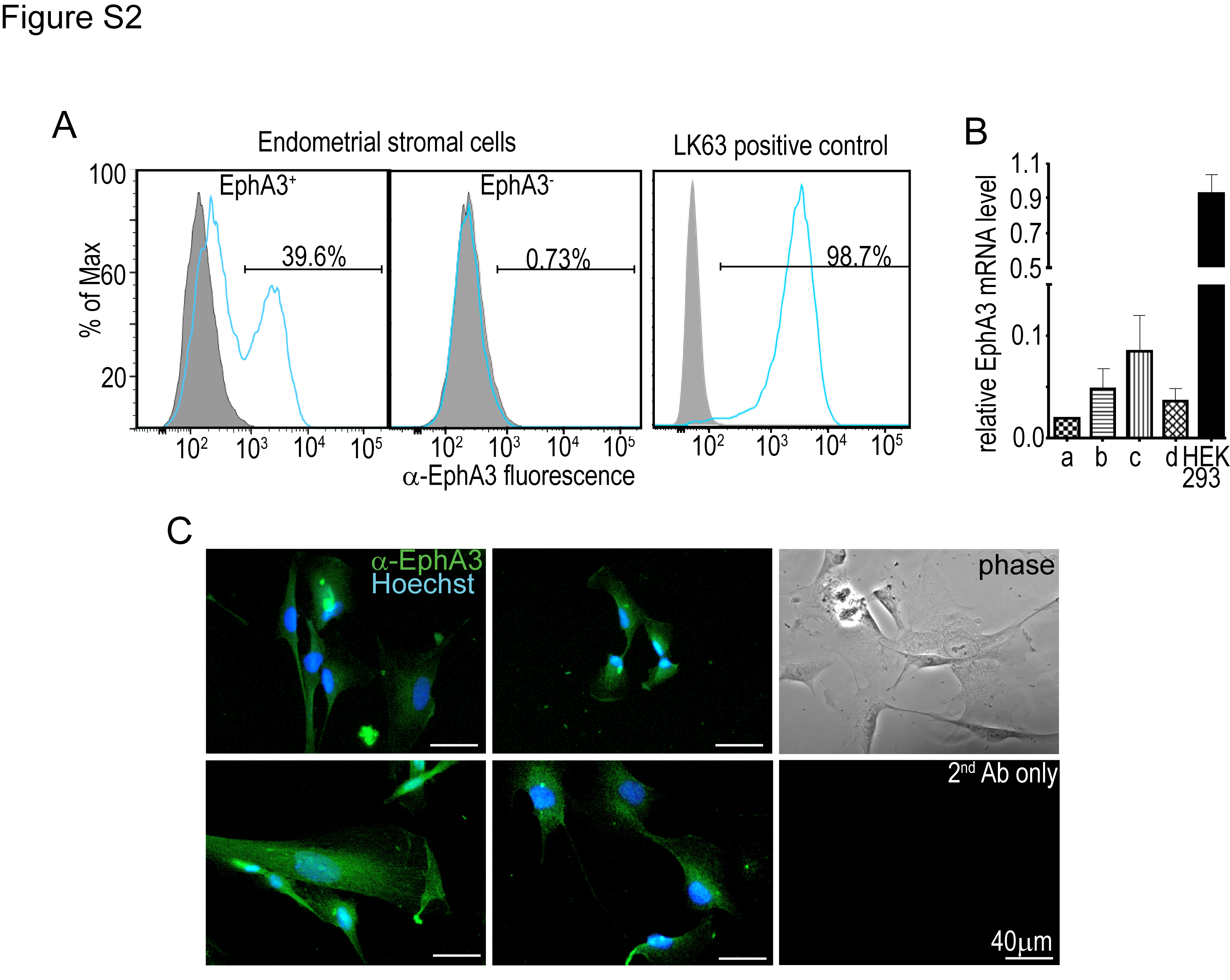

Supplement: Figure S2 — EphA3 expression in human endometrial stromal cells. (A) Flow cytometry of endometrial stromal cells (eSCs) fractionated by MACS into EphA3+ (example shown after two rounds of isolation) and EphA3-depleted (EphA3-) eSCs. LK63 pre-B leukemic cells serve as a positive control for binding of the IIIA4 α-EphA3 antibody. Shaded peak indicates isotype control-stained cells. (B) qRT-PCR of several different EphA3+eSC preparations (a–d) at passage 3, relative to β-actin expression. HEK293 cells were used as a control cell line with established EphA3 expression. (C) Fluorescence microscopy of MACS-isolated EphA3+ eSCs from different preparations; PFA-fixed and permeabilised cells were stained with rabbit α-EphA3 antibodies, Alexa488-conjugated secondary antibodies and Hoechst nuclear stain. Fluorescent (2nd Ab only) and phase contrast (phase) micrographs of cells stained with Alexa488-labelled secondary antibodies are shown as controls, scale bars: 40 µm. (TIF) [file pone.0112106.s002.tif]

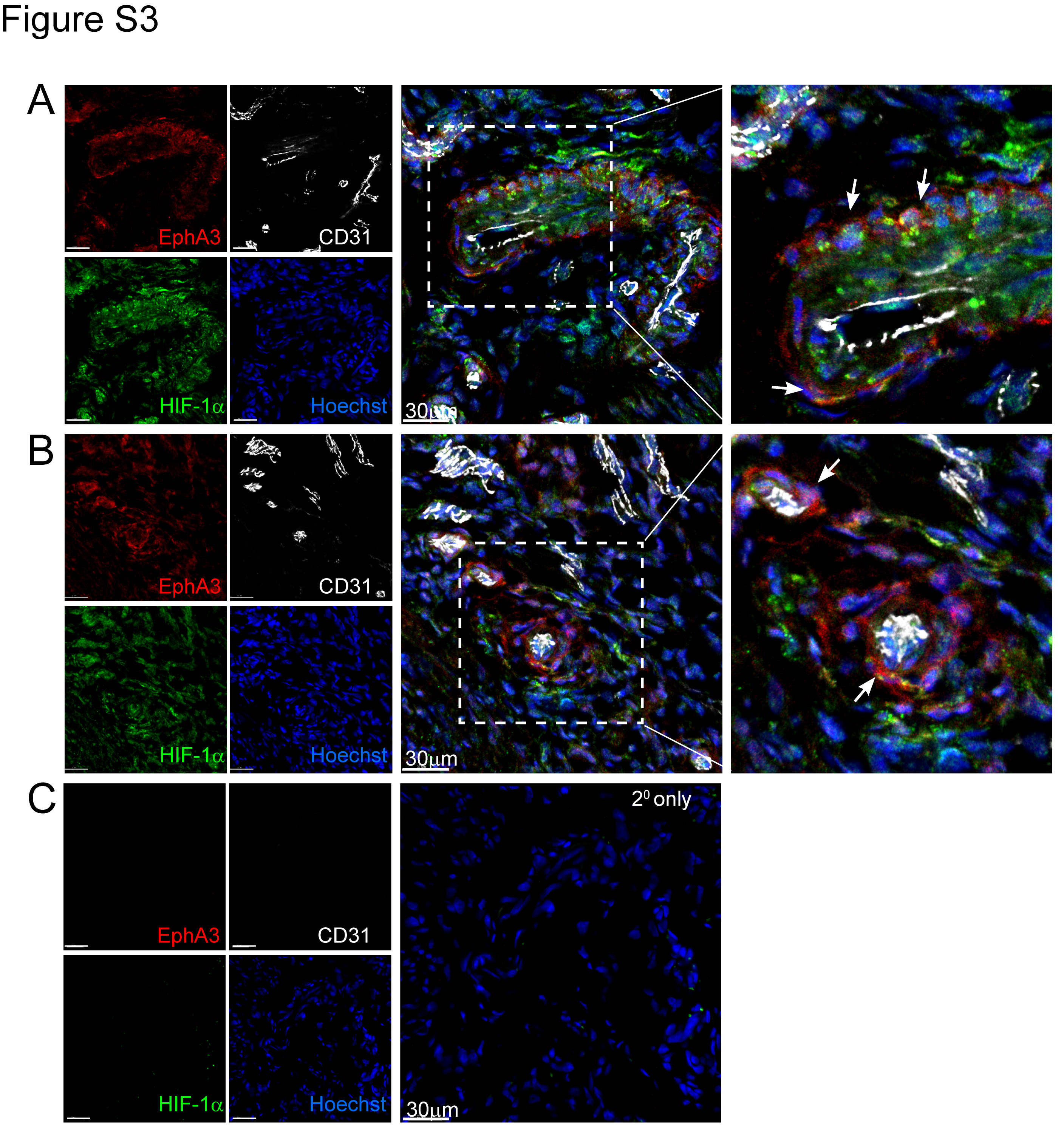

Supplement: Figure S3 — Immunofluorescence detection of HIF-1α in human endometrium. Frozen sections of secretory-phase human endometrium were immunostained for EphA3 (red) and HIF-1α (green), along with CD31 antibodies to mark endothelial cells (white) and Hoechst to stain nuclei (blue). Boxed sections are shown magnified 2x in the panels to the right. Arrows indicate EphA3/HIF-1α co-staining in perivascular cells. Results are representative of n = 6 independent samples. Examples shown are: (A) a large vessel in the basal layer; (B) smaller spiral arterioles in the functional layer; (C) secondary antibodies only as negative control. Scale bar: 30 µm. (TIF) [file pone.0112106.s003.tif]

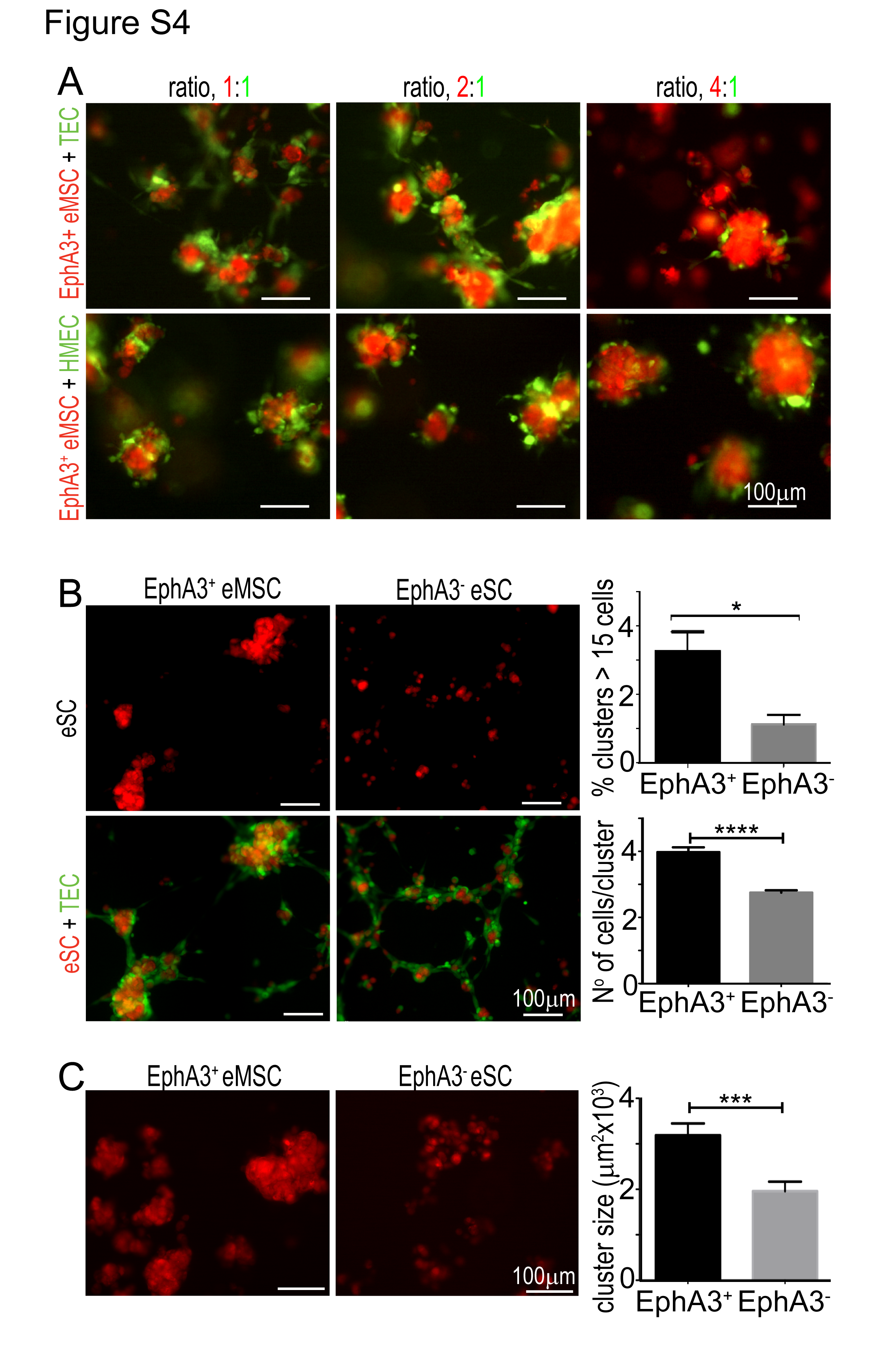

Supplement: Figure S4 — EphA3+eMSCs promote the assembly of MSC/endothelial cell organoids. (A) The assembly of 3D cell clusters from EphA3+eMSC (red) and tumour endothelial cells (TECs) or human microvascular endothelial cells (HMEC; green) at indicated cell ratios was analysed in overnight co-cultures in growth-factor-reduced Matrigel. Independent of cellular ratios, TECs and HMECs interact with eSCs by forming an outer cell layer around a central eSC cluster. (B) 3D eSC/endothelial cell clusters from 1∶2 ratios of EphA3+eMSC (EphA3+) or EphA3-depleted (EphA3-) eSC and TECs. While TECs interacted with both stromal cell populations, EphA3+eMSCs revealed significantly increased frequency of forming larger organoids. Mean and SE are shown, * p<0.05 (Student's t-test); **** p>0.0001 (Kruskall-Wallis test). (C) Assembly into large cell clusters is independent of endothelial cells. Images depict overnight cultures of EphA3+ eMSCs and EphA3-depleted eSC (EphA3-) alone on growth-factor-reduced Matrigel. Mean and SE are shown; *** p = 0.0003 (Kruskall-Wallis test). Representative fields of views are illustrated in all images (A–C), all indicated scale bars are 100 µm. (TIF) [file pone.0112106.s004.tif]

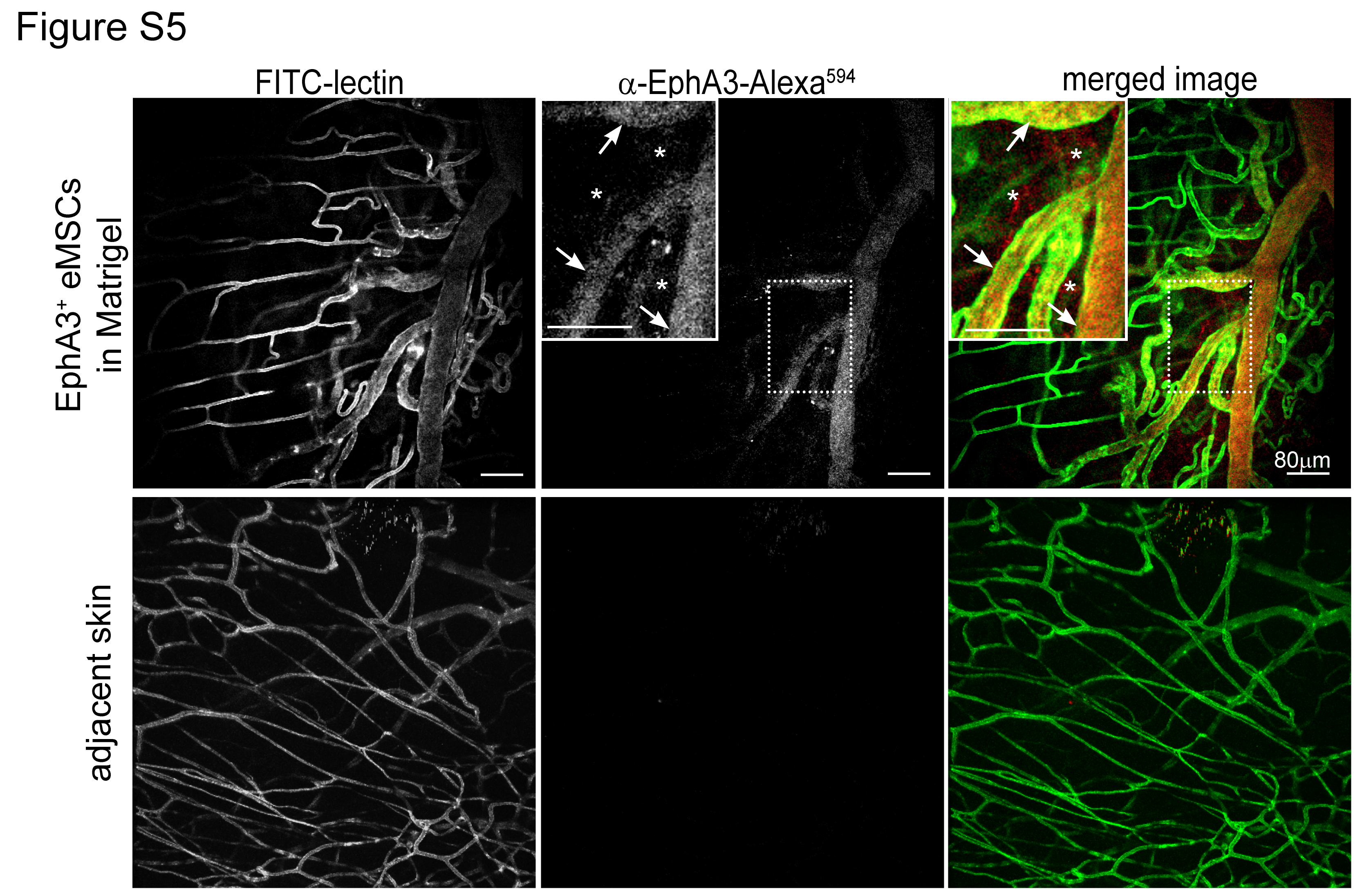

Supplement: Figure S5 — Localisation of fluorescently-labelled α-EphA3 antibodies to growing blood vessels in vivo . Intravital 2-photon microscopy of subcutaneous Matrigel plugs with EphA3+eMSCs, 5 weeks after implantation. Mice had been injected intravenously with Alexa594IIIA4 to detect EphA3 expression (red) and [FITC]RCA-lectin to delineate blood vessels (green). Normal skin sections adjacent to the Matrigel (bottom panels) were imaged as controls. Individual and merged fluorescent channels are shown; 80 µm scale bars. Arrowheads indicate EphA3+ vessels; stars indicate IIIA4-stained perivascular stromal tissue. (TIF) [file pone.0112106.s005.tif]

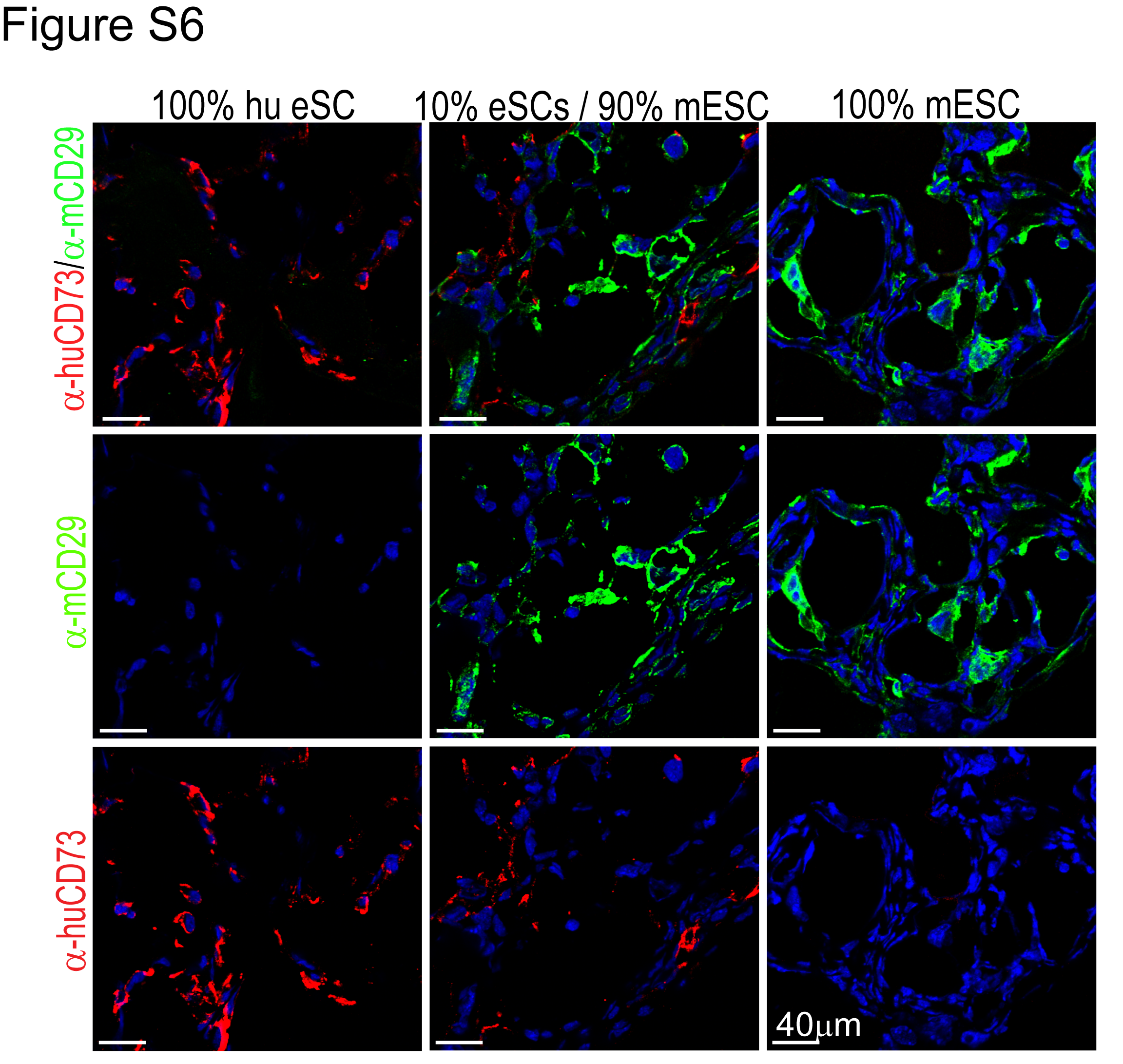

Supplement: Figure S6 — Species-specific detection of human eSCs. The species specificity of the α-human CD73 antibody used to detect transplanted EphA3+eMSCs in mice was validated using frozen sections of fibrin cell clots. Clots composed of 100% unsorted human (hu) eSCs, 100% mouse embryonic stem cells (mESCs), or 90% mESC/10% human eSC were sectioned and immunostained with a combination of α-human CD73 (red) and α-mouse CD29 (green) antibodies. Staining of human or mouse cells with these antibodies is non-overlapping. Scale bar: 40 µm. (TIF) [file pone.0112106.s006.tif]
