# Supplementary material for: Hypoxia-Controlled EphA3 Marks a Human Endometrium-Derived Multipotent Mesenchymal Stromal Cell that Supports Vascular Growth
Source: PLoS One. 2014 Nov 24;9(11):e112106. doi: 10.1371/journal.pone.0112106 (PMC4242616; doi:10.1371/journal.pone.0112106)
Supplement: Table S1 — qRT-PCR primer sequences. (DOCX) [file pone.0112106.s007.docx]

**Table S1: qRT-PCR primer sequences.**

|  | **qRT-PCR primers** | |
| --- | --- | --- |
|  | **Forward 5’-3’** | **Reverse 5’-3’** |
| **18S** | GTAACCCGTTGAACCCCATG | CCATCCAATCGGTAGTAGCG |
| **b-actin** | TTGTTACAGGAAGTCCCTTGCC | ATGCTATCACCTCCCCTGTGTG |
| **EphA2** | GGGACCTGATGCAGAACATC | AGTTGGTGCGGAGCCAGT |
| **EphA3** | AAACTAGTCCAGACTCTTTCT | ACCTCCCAATCAAAACATAGATG |
| **EphB2** | CCTGCCTCGAGGACTGATACTG | ATGCAGGATCAACCCTTCTCA |
| **EphB3** | CGCTCAGTCTGGCATGTCA | CATCCAGCCAATCACCAACTG |
| **EphB4** | TGTGTTGGAGGGAACCTGTTTC | GGGCCCCTGTTTCAACTTG |
| **ephrinA1** | CCGGAGAAGCTGTCTGAGAA | GGTTTGGAGATGTAGTAGTAGCTGTG |
| **ephrinA2** | CTCGGAGAAGTTCCAGCTC | GGCGTGGCAGAGATGTAGTA |
| **ephrinA3** | GAGGATGAAGGTGTTCGTCTG | CCCTCAAAGTCTTCCAGCAC |
| **ephrinA4** | TGGCCATGTTCAATTCTCAG | AGTGGGCACCGAGATGTAG |
| **ephrinA5** | TTCTACATCTCCTCTGCAATCC | ACAGCTATTTGTTGGTCTCACA |
| **ephrinB2** | GCAAGTTCTGCTGGATCAAC | CAAAGGGACTTGTTGTCGAA |
| **CD106** | ACTTGATGTTCAAGGAAGAG | TCCAGTTGAACATATCAAGC |
| **KDR** | GTACATAGTTGTCGTTGTAGG | TCAATCCCCACATTTAGTTCCTGATA |
| **CD13** | AGGGCCTGGCGTCCTAC | GTGGTGGGGAGTTGGATGG |
| **PDGFRα** | TCAAGTTCCTTCATCCATTC | CATCCACTCAATATCAGGAAG |
| **CD10** | CTGATATCAACACTCCAAAGC | TCATCGTAGGTTGCATAGAG |
| **CD73** | ATAAGGTGATCCTCCCAAAC | ATATCTTGGTCACCAGAGTC |
| **CD44** | GGCCTTGGCTTTGATTCTTGC | GAGACTTGCTGGCCTCTCC |
| **CD29** | ATTCATGACAGAAGGGAGTTTGC | GTGTTGTGGGATTTGCACGG |
| **CD49f** | CCAATTCGTCTTAATGGAACC | ATATCTGGGTAGCCATCTTG |
| **PDGFRβ** | GGGAAGAGAAGTTTGAGATTC | TTCTTTTTGTAACCTTCGCC |
| **CD90** | CAGTGCTCAGAGACAAAC | CAGCTGACTCAGAGAAGTAG |
| **CD105** | CAAGTCTTGCAGAAACAGTC | TAGTGGTATATGTCACCTCG |
| **CD146** | TCTGTCCACAAGGAGAAGC | CCTCCGGAGCTTTGTAGAC |
| **NG2** | CCACTCAGCAGTCAGACCC | GCTCATACAGAATATTCCCAGCG |
