# Supplementary material for: Hypoxia-Controlled EphA3 Marks a Human Endometrium-Derived Multipotent Mesenchymal Stromal Cell that Supports Vascular Growth
Source: PLoS One. 2014 Nov 24;9(11):e112106. doi: 10.1371/journal.pone.0112106 (PMC4242616; doi:10.1371/journal.pone.0112106)
Supplement: Table S2 — mRNA expression profile of Ephs and ephrins in EphA3+eSCs. The mRNA expression levels of indicated Ephs and ephrins (epn) in EphA3+eSCs, cultured under γnormoxic (20% O2) or ‡hypoxic (1% O2) conditions, were determined by qRT-PCR. *The gene with the lowest mRNA expression is designated as calibrator (ephrinA1) with the value 1.0. §Fold relative changes of mRNA levels under hypoxic versus normoxic conditions for the indicated Ephs and ephrins are shown. (DOCX) [file pone.0112106.s008.docx]

**Table S2:** **mRNA expression profile of Ephs and ephrins in EphA3^+^eSCs.**

|  | **EphA2** | **EphA3** | **EphB2** | **EphB4** | **^*^epnA1** | **epnA3** | **epnA4** | **epnA5** | **epnB2** |
| --- | --- | --- | --- | --- | --- | --- | --- | --- | --- |
| **^γ^20% O_2_**  **^‡^1% O_2_**  **^§^Change** | 34551.8  8159.4  ↓4.2 | 9.4  29.8  ↑3.2 | 121.6  26.1  ↓4.7 | 1291.2  402.1  ↓3.2 | 1.0  3.3  ↑3.4 | 35.6  254.8  ↑7.2 | 356.0  276.5  ↓0.8 | 85.9  51.7  ↓1.3 | 712.8  450.2  ↓1.6 |

The mRNA expression levels of indicated Ephs and ephrins (epn) in EphA3^+^eSCs, cultured under ^γ^normoxic (20% O_2_) or ^‡^hypoxic (1% O_2_) conditions, were determined by qRT-PCR. ^*^The gene with the lowest mRNA expression is designated as calibrator (ephrinA1) with the value 1.0. ^§^Fold relative changes of mRNA levels under hypoxic versus normoxic conditions for the indicated Ephs and ephrins are shown.
